# Radiomics Models for Predicting Microvascular Invasion in Hepatocellular Carcinoma: A Systematic Review and Radiomics Quality Score Assessment

**DOI:** 10.3390/cancers13225864

**Published:** 2021-11-22

**Authors:** Qiang Wang, Changfeng Li, Jiaxing Zhang, Xiaojun Hu, Yingfang Fan, Kuansheng Ma, Ernesto Sparrelid, Torkel B. Brismar

**Affiliations:** 1 Division of Medical Imaging and Technology, Department of Clinical Science, Intervention and Technology (CLINTEC), Karolinska Institutet, 14186 Stockholm, Sweden; torkel.brismar@ki.se; 2 Division of Radiology, Department of Clinical Science, Intervention and Technology (CLINTEC), Karolinska Institutet, Karolinska University Hospital, 14186 Stockholm, Sweden; 3 Institute of Hepatobiliary Surgery, Southwest Hospital, Army Medical University (Third Military Medical University), Chongqing 400038, China; changfeng.li2020@outlook.com (C.L.); kuanshengma@outlook.com (K.M.); 4 Department of Pharmacy, Guizhou Provincial People’s Hospital, Guiyang 550002, China; zjx19870619@126.com; 5 Hepatobiliary Surgery, The Fifth Affiliated Hospital, Southern Medical University, Guangzhou 510999, China; huxj2016@163.com; 6 The Second School of Clinical Medicine, Southern Medical University, Guangzhou 510515, China; fanxifan@smu.edu.cn; 7 Hepatobiliary Surgery, Zhujiang Hospital, Southern Medical University, Guangzhou 510280, China; 8 Division of Surgery, Department of Clinical Science, Intervention and Technology (CLINTEC), Karolinska Institutet, Karolinska University Hospital, 14186 Stockholm, Sweden; ernesto.sparrelid@ki.se

**Keywords:** radiomics, microvascular invasion, primary liver cancer, prediction model, systematic review

## Abstract

**Simple Summary:**

Microvascular invasion (MVI) is regarded as a sign of early metastasis in liver cancer and can be only diagnosed by a histopathology exam in the resected specimen. Preoperative prediction of MVI status may exert an effect on patient treatment management, for instance, to expand the resection margin. Radiomics can identify delicate imaging features from routinely used radiological images that are invisible to the naked eye and has been increasingly adopted to predict MVI. We reviewed the available radiomics models to evaluate their role in the prediction of MVI. The discriminative capacity of the models ranged from 0.69 to 0.94. Even though the studies were preliminary and the methodologic quality was suboptimal, radiomics models hold promise for the accurate and non-invasive prediction of MVI. In accordance with a standardized radiomics workflow, future prospective studies with external validation are expected to provide a reliable and robust prediction tool for clinical implementation.

**Abstract:**

Preoperative prediction of microvascular invasion (MVI) is of importance in hepatocellular carcinoma (HCC) patient treatment management. Plenty of radiomics models for MVI prediction have been proposed. This study aimed to elucidate the role of radiomics models in the prediction of MVI and to evaluate their methodological quality. The methodological quality was assessed by the Radiomics Quality Score (RQS), and the risk of bias was evaluated by the Quality Assessment of Diagnostic Accuracy Studies (QUADAS-2). Twenty-two studies using CT, MRI, or PET/CT for MVI prediction were included. All were retrospective studies, and only two had an external validation cohort. The AUC values of the prediction models ranged from 0.69 to 0.94 in the test cohort. Substantial methodological heterogeneity existed, and the methodological quality was low, with an average RQS score of 10 (28% of the total). Most studies demonstrated a low or unclear risk of bias in the domains of QUADAS-2. In conclusion, a radiomics model could be an accurate and effective tool for MVI prediction in HCC patients, although the methodological quality has so far been insufficient. Future prospective studies with an external validation cohort in accordance with a standardized radiomics workflow are expected to supply a reliable model that translates into clinical utilization.

## 1. Introduction

Microvascular invasion (MVI) has been recognized as an independent predictor for early recurrence and poor prognosis after liver resection or transplantation in hepatocellular carcinoma (HCC) [1,2]. Its reported incidence ranges from 15% to 57% according to different diagnostic criteria and study population [3]. The diagnosis of MVI, however, is only made by a postoperative histopathology exam on the resected specimen, which exerts little or no influence on the patient treatment management, while with the knowledge of MVI, clinicians can optimize a patient treatment strategy, for example, to expand the resection margin in operation or to adopt an alternative treatment option. To implement personalized medicine, it is of utmost importance to preoperatively identify and stratify patients with MVI. Therefore, a reliable, noninvasive biomarker for preoperative prediction of MVI is urgently needed.

Medical imaging has evolved from a primarily diagnostic tool to an essential role in clinical decision making. Clinically, radiologists use pattern recognition after establishing links between radiological features at CT or MRI images and MVI [4,5], such as arterial peritumoral enhancement, non-smooth tumor margins, and rim arterial enhancement [2]. The Liver Imaging Reporting and Data System (LI-RADS) has recently been developed and has evolved as a comprehensive and standardized diagnostic algorithm for HCC imaging reporting [6]. LI-RADS has been proven to be an effective tool not only for HCC diagnosis but also for outcome prediction after liver resection, radiofrequency ablation, or liver transplantation [6,7,8], exerting an increasing influence on the treatment management of HCC. Previous studies have demonstrated the diagnostic value of LI-RADS in the prediction of MVI [9,10]. However, these qualitative features suffer from their subjectivity and high inter-observer variability [11].

Radiomics is an emerging field that can extract high-throughput imaging features from biomedical images and convert them into mineable data for quantitative analysis [12,13]. Its basic assumption lies on that the alterations and heterogeneity of the tumor on the micro scale (e.g., cell or molecular levels) can be reflected in the images [14]. Therefore, through radiomics analysis, the cancerous cell emboli (i.e., MVI) in the hepatic vasculature can be detected in the preoperative images, which holds promise for the preoperative prediction of MVI and personalized treatment. In recent years, a number of radiomics models for MVI prediction have emerged. However, there has not been any research systematically summarizing current radiomics research for MVI prediction, and the overall efficacy of the prediction model is still unknown. In addition, as radiomics research is a sophisticated process and consists of several steps, it is important to evaluate the methodological variability to obtain a reliable and reproducible model before translating it to clinical applications. The current systematic review therefore aims (1) to provide an overview of radiomics studies for MVI prediction in HCC patients and assess the efficacy of the prediction models and (2) to evaluate the methodologic quality in the radiomics workflow and the risk of bias in the research.

## 2. Materials and Methods

This study is registered at the PROSPERO website (https://www.crd.york.ac.uk/prospero/, No: CRD42021250082, (accessed on 20 May 2021)) and was conducted under the guidance of the Preferred Reporting Items for a Systematic Review and Meta-analysis of Diagnostic Test Accuracy Studies (PRISMA-DTA) (Table S1).

### 2.1. Literature Research and Study Selection

Publications from databases of the PubMed, Embase, Web of Science, and Cochrane libraries were systematically retrieved by using the following key terms: “radiomics/texture analysis”, “microvascular invasion”, and “hepatocellular carcinoma”. Detailed searching queries in each database can be found in Table S2. The last updated date of the literature search is 29 May 2021.

Records satisfying the following criteria were considered as eligible: Inclusion criteria: (1) retrospective or prospective studies; (2) studies considering patients who were diagnosed with hepatocellular carcinoma by a pathology exam; (3) studies with radiomics features extracted from CT, MRI, or PET/CT images used as predictors for MVI, solely or as a variable in a model; (4) studies where MVI prediction is the main outcome or one of the main outcomes; (5) publications in English. Exclusion criteria: (1) publications in the form of a letter, conference abstract, editorial, review, or case report; (2) research considering only semantic radiological features used for MVI prediction; (3) research with operator-dependent imaging modalities, such as ultrasound-based studies; (4) deep-learning research not involving any textural features in the model; (5) studies only evaluating the predictive value of a single radiomics feature, without any combination into a multiple features prediction model; (6) studies with a sample size of less than 30.

Study selection was conducted by two reviewers (Q.W. and C.L.) by screening the title and abstract and then the full text. Any disagreement or uncertainty was resolved by two senior researchers (K.M. and T.B.) to reach a consensus. Reference lists of the enrolled studies as well as a pre-existing systematic review/meta-analysis were also searched manually to recruit any potentially eligible studies.

### 2.2. Data Extraction

**Figure 1:**
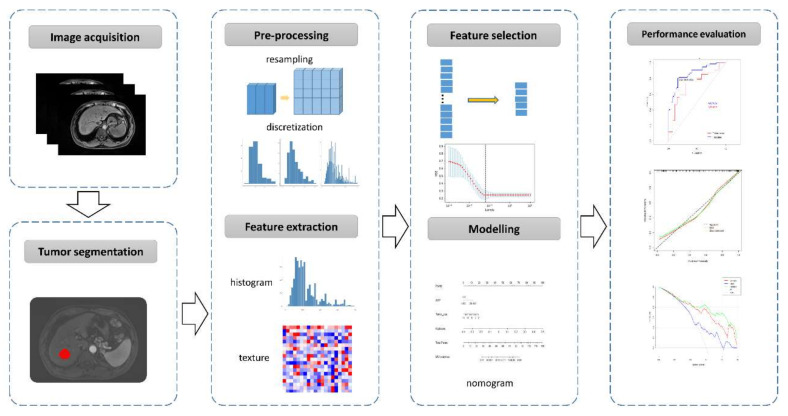
A typical workflow of radiomics research for microvascular invasion (MVI) prediction in hepatocellular carcinoma.

A pre-defined table was used to extract the following information from each paper: (1) general study characteristics; (2) patient characteristics; (3) characteristics in development of a radiomics model, including imaging modalities, tumor segmentation, imaging preprocessing and feature extraction, and feature selection and modelling; (4) performance metrics of a radiomics model, including area under the receiver operating characteristic (ROC) curve (AUC), calibration statistics, and decision analysis. A typical radiomics research workflow for MVI prediction is illustrated in Figure 1.

If several prediction models were developed in one study, the one with the best performance in the test cohort was selected. For studies from the same medical center with subjects overlapped, if the same imaging modality was adopted, the latest study was included; if different modalities or different contrast media used in the same modality were applied, both studies were enrolled. Supplemental files of included studies were also screened to extract required data, if necessary.

The terms “test cohort” and “validation cohort” were unified in this study to avoid potential misunderstanding and confusion. A “test cohort” is a part of the model development cohort and usually refers to an “internal test cohort”. A “validation cohort” is independent from the model development cohort, be it temporal validation (data collected from a later period) or geographic validation (data sampled from another hospital or country) [15], and it is often called an “external validation cohort”.

### 2.3. Assessment of Radiomics Quality Score, Risk of Bias, and Research Type

The Radiomics Quality Score (RQS) is a scoring system proposed by Lambin in 2017 [16] and is commonly used for evaluating the methodologic quality of the radiomics research [17,18]. The RQS tool contains 16 key items to quantify the quality of the radiomics workflow and the reporting. Most items are designated to 0, 1, or 2 points, according to how well a study achieves the signaling question. To highlight the importance of some dimensions, a higher point is assigned; for example, 7 points is given to a prospective validation study, and 5 points is given to a study validated in three or more datasets. The ideal score of the RQS is 36 points, responding to a percentage of 100%. Table S3 provides a detailed description of the RQS items.

As the radiomics model is also used as a diagnostic tool, the risk of bias and the applicability concerns of the included studies were further assessed by using the revised Quality Assessment of Diagnostic Accuracy Studies (QUADAS-2) tool [19]. QUADAS-2 evaluates the risk of bias of a study in four domains: patient selection, index test, reference standard, and flow and timing. The results of each domain were marked as low, high risk, or unclear. Detailed description of QUADAS-2 is provided in Table S4.

An assessment of the RQS and QUADAS-2 was independently performed and cross-validated by two reviewers (Q.W. and C.L.). When discrepancy occurred, agreement was reached after discussion with two senior researchers (K.M. and T.B.).

## 3. Results

### 3.1. Literature Selection

**Figure 2:**
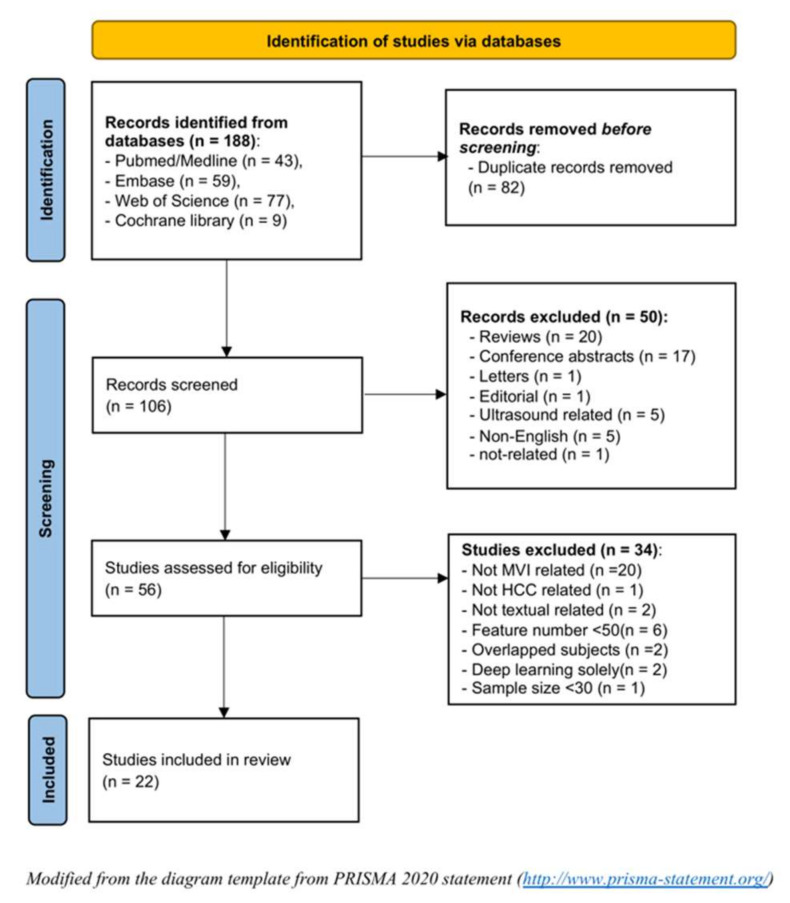
Flow chart of the study selection.

The systematic literature search initially yielded 188 records from the four databases. After removing 82 duplicates, 50 inappropriate types of publications, and 34 ineligible studies, a total of 22 studies were included in this systematic review [20,21,22,23,24,25,26,27,28,29,30,31,32,33,34,35,36,37,38,39,40,41] (Figure 2).

### 3.2. General Characteristics and the Incidence of MVI

The included 22 studies were published between September 2017 and May 2021, with two thirds (15/22) within the last two years. All studies were retrospectively designed and, in total, included 5552 patients with a sample size varying from 69 to 637 patients (median: 174). Most studies (20/22) split the cohort into a training and a test cohort, while only two of them further validated their model using an independent external cohort [25,29]. Nine studies (8/22) focused on solitary HCC, among which five focused on HCC with a diameter of less than 5 cm.

The incidence of MVI ranged from 25.3% to 67.5% for an individual entire cohort, and 25.3% to 56.4% for HCC less than 5 cm. Around two thirds (16/22) of the studies explicitly stated their definition of MVI. Table 1 gives more details about the general characteristics of the reviewed studies.

### 3.3. RQS and Risk of Bias Assessment

**Figure 3:**
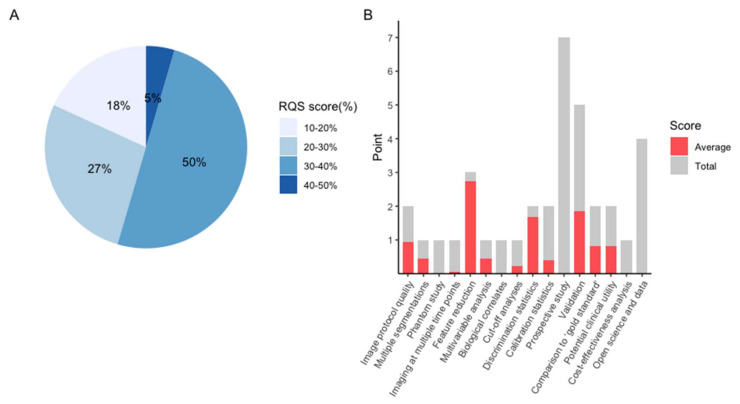
Methodological quality evaluated by using the Radiomics Quality Score (RQS) tool. (**A**). Proportion of studies with different RQS percentage score. (**B**). Average scores of each RQS item (gray bars stand for the full points of each item, and red bars show actual points).

The average RQS score of the included studies was 10, accounting for 28% of the total points. The highest RQS score was 15 points (42%), seen in only one study [16]. Around half of the studies were credited between 11 and 14 points, corresponding to 30–40% of total points (Figure 3A). As no research considered the items of “phantom study”, “prospective study”, “detect and discuss biological correlates”, “cost-effectiveness analysis”, or “open science and data”, these five items were assigned 0 points. Other poorly performed items include “imaging at multiple time points”, “cut-off analysis”, and “calibration statistics”, in which the average points for each item were less than 30% (Figure 3B). A detailed description and a summary of the RQS score are provided in Table S5.

**Figure 4:**
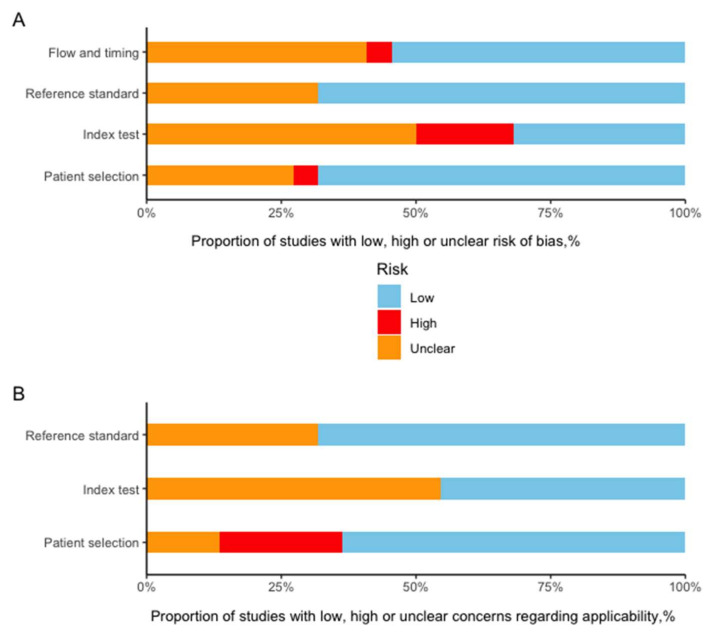
Grouped bar charts of the risk of bias (**A**) and applicability concerns (**B**) of the included studies assessed by using a revised tool for the Quality Assessment of Diagnostic Accuracy Studies (QUADAS-2).

The summary of the risk of bias and the applicability concerns evaluated by the QUADAS-2 tool are presented in Figure 4. Most studies showed a low or unclear risk of bias in each domain. Detailed description in each domain is provided in Table S6.

### 3.4. Study Characteristics

According to the typical radiomics workflow, the study characteristics is summarized as follows.

#### 3.4.1. Imaging Acquisition

CT was applied in 10 studies, MRI in 10 studies, and both modalities in 1 [40], and only 1 used PET/CT [34]. Most studies (16/22) exploited more than one phase/sequence to construct their prediction model. The interval between the preoperative imaging exam and liver resection (for histopathological diagnosis of MVI) varied from 1 week to 3 months (median: 1 month).

#### 3.4.2. Tumor Segmentation

A majority of studies performed 3D segmentation (20/22). In 15 of these studies, 3D segmentation was achieved manually; in 3, segmentation was semi-automatic [21,28,34]; in the remaining 2, it was achieved automatically [20,31]. Two studies manually delineated the tumor on the cross-section slice with the largest tumor diameter [24,28]. Nine studies expanded the segmented tumor with different distances, and the most common dilated distance was 10 mm from the tumor margin.

#### 3.4.3. Imaging Preprocessing and Feature Extraction

As imaging may come from different centers, different manufacturers, and different scanners, imaging preprocessing prior to feature extraction is necessary to increase the reliability of the textural measurements. Six studies (6/22) resampled the images before feature extraction, most often to a voxel size of 1 × 1 × 1 mm^3^.

The most commonly used software to extract imaging features was pyradiomics (9/22), followed by MatLab or its related software (6/22). The number of radiomics features extracted from each phase/sequence ranged from 58 to 2932.

#### 3.4.4. Feature Selection and Modelling

To avoid potential overfitting during development of a radiomics model, feature selection and dimensionality reduction is necessary, as the radiomics features often outnumbered the sample size. The most widely used algorithm was the Least Absolute Shrinkage and Selection Operator (LASSO) regression, which is an efficient method to select informative variables by introducing L1 regularization (15/22). The number of imaging features included in the radiomics model ranged from 2 to 74 (median: 15), and the event/feature ratio ranged from 0.7 to 35.5 (median: 4.2). Nine studies further included clinical risk factors into a combined prediction model. High alpha-fetoprotein (AFP) (9/22) and a large tumor size (4/22) were both frequently detected clinical risk factors for MVI prediction.

It is worth mentioning that the reproducibility evaluation of imaging features can also be used for feature selection. Among the 10 studies that performed interclass correlation coefficient (ICC) analysis, 4 of them set a threshold of 0.8 for robust features and selected those for further analysis [32,38,40,41].

#### 3.4.5. Performance of the Prediction Model

A majority of studies (20/22) split the subjects into training and test subsets. The median AUC in the test cohort was 0.79, ranging from 0.69 to 0.94. Two studies validated their models using an independent cohort with AUCs of 0.84 and 0.80 [29,31]. Only five studies reported the cut-off value when presenting the performance metrics [21,26,32,34,41]. Nine studies (9/22) evaluated the calibrated ability of their model in the form of a calibration curve and clinical usefulness of the model in the form of decision curve analysis.

The abovementioned characteristics of the radiomics workflow has been provided in detail in Table 2 and Table S7.

## 4. Discussion

The present study identified an ever-growing number of studies performing radiomics analysis of HCC for MVI prediction, mostly published in the last two years. The added value of radiomics in imaging modalities used in clinical routines has been explored extensively, with an AUC as high as 0.80–0.84 in two independent validation cohorts, shedding light on the management and prognosis prediction of HCC patients. Although the initial results are promising and encouraging, the methodological variability of the research is considerable, and the reporting quality is insufficient. Before translating the radiomics model to clinical applications, it is urgent to standardize the reporting norms to make the prediction models reproducible and reliable and to validate the models in external cohorts.

Radiomics research is a complex, interdisciplinary, multi-step project, involving image processing, big data handling, algorithm operating, model construction, and validation. Each step in the radiomics workflow can be achieved by several different strategies and approaches, which induces substantial methodological heterogeneity among radiomics studies. The variability started with different imaging modalities, followed by different tumor segmentation strategies and different categories of radiomics features, as well as different algorithms and classifiers used for feature selection and modelling. Moreover, variability existed even in the same imaging modality; e.g., MRI acquisition may vary in terms of the manufacturers, scanning protocols, contrast media, and sequence/phase used, and the various software and tools applied for feature extraction inevitably resulted in radiomics features with different nomenclatures. Therefore, it seems hard to pool data across studies and to enable a robust meta-analysis. Given that the radiomics workflow involves multiple steps, it poses a challenge for other researchers to reproduce findings when the original study does not supply sufficient detail. Instead, improving the reporting quality seems to be a practical approach to validating the findings and translating them into clinical utility. However, the present review has highlighted the insufficient reporting quality of current radiomics HCC-MVI research, which was reflected by an average RQS score of 10 (28% of the total points). This finding is similar to the result of a recent systematic review that evaluated radiomics research quality in the area of HCC, with a mean RQS score of 8.4 [42].

Five items of the RQS in which all included studies performed poorly are “prospective study”, “phantom study”, “biological correlates”, “cost-effectiveness analysis”, and “openness of data and code”. A well-designed prospective study can reduce and minimize the potential confounding factors, representing a higher level of evidence for the quality validity. Thus, prospective studies are given the highest weighting in the RQS tool (7 points, accounting for around 20% of the full scale). However, to date, no prospective radiomics MVI research has been performed. A phantom study’s purpose is to detect potential feature variability among different scanners and manufacturers. This is of great importance, as the evaluated cohorts often involve many scanners or even different medical centers. The phantom study process ensures that only robust features are included in the following radiomics analysis. Biological correlates aim to link imaging findings with gene or molecular signatures. However, none of the reviewed studies evaluated the gene or molecular levels of the tumor samples. Previous studies have detected a 91-gene signature that highly correlates with vascular invasion in HCC [43]. Based on this finding, a contrast-enhanced CT imaging biomarker, i.e., radiogenomic venous invasion (RVI), which includes three imaging features (internal arteries, a hypo-dense halo, and a tumor-liver difference), has been shown to be an accurate predictor of MVI [44]. Future studies are required to explore and verify the correlations between radiomics features and gene expressions. A cost-effectiveness analysis can evaluate a radiomics prediction model in terms of health economics when applied in clinical routines. It assumes that a novel predictor should not be more expensive than currently available predictors when accuracy is comparable. It also compares the health effect of a radiomics predictor with a condition without a radiomics predictor, such as a quality-adjusted life year analysis. We think that evaluating this point seems less urgent, given that the methodological standardization and clinical/biological validation of current radiomics models are still lacking. Data and code openness aims to repeat and reproduce results and findings and to further validate and promote the prediction model in other centers. Though some initiatives have been proposed in an attempt to remove the obstacles in data sharing, other factors, such as legal/privacy issues, culture/language barriers, and insufficient staff/time, still exist [10]. None of the studies shared their codes or imaging data publicly.

Regarding the items of “imaging at multiple time points” and “multiple segmentations”, both aim to select stable imaging features for modelling considering subjective and temporal variations. However, less than half of the studies performed ICC analysis and seldom explicitly stated that imaging features from different phases/sequences were evaluated during that analysis (i.e., test–retest analysis). Furthermore, there is no generally accepted ICC threshold at which radiomics features can be considered robust. Generally, when reporting ICC, values of 0.75–0.90 are regarded as indicating good reliability, and values higher than 0.9 are regarded as excellent [45]. However, among the studies that calculated ICC, the applied threshold varied among 0.75, 0.80, and 0.9. A future study should be applied to determine the proper threshold at which robust radiomics features for modelling can be defined. Interestingly, some of the studies reported here did not rule out features with low ICC and constructed their model using only the full features extracted from their images.

When evaluating the performance and clinical utility of the radiomics model considering the items of “cut-off analysis”, “calibration statistics”, “comparison with gold-standard”, “potential clinical utility”, and “validation”, the included studies again were insufficient. The performance metrics of a model, such as the sensitivity and specificity, are often determined by a specified cut-off value, and this value can further classify a patient cohort into high and low risk groups for a certain condition. A cut-off value is also one of the prerequisites for reproducing the results of previous research. However, only five studies reported their cut-off values. Regarding calibration analysis, which evaluates the agreement between predictions and the actual events, less than half of the studies performed one. Regarding the comparison with “gold-standard”, there is currently no surrogate that can serve as a “gold-standard” for MVI prediction. As the value of semantic imaging features have been extensively explored for MVI prediction, we therefore defined conventional imaging features as the “gold-standard”. Among the 10 studies that compared prediction performance between radiomics and radiologist models, all declared that the radiomics models outperformed the radiologists’ semantic models (Table S7). However, the publishing bias should be borne in mind when interpreting these results. Only two studies validated their models using independent external cohorts. However, one of them validated their model in only 18 patients, which is not a sufficiently large validation cohort according to the “10-EPV” principle (at least 10 events per variable) [16,46]. When developing a prediction model, the ratio of event and variable should be maintained at a certain level to avoid potential overfitting or underfitting. Among the 16 studies with an EPV ratio available, the median EPV (MVI positive cases/features) ratio was 4.2, indicating a potential risk of overfitting. Therefore, it is assumed that, before translating these models into a clinical routine utility, some practical issues should be well addressed, such as the reproducibility of the radiomics model, the standardization of imaging protocols, model overfitting, and the external validation of the prediction models.

Though the RQS tool aims for high-quality radiomics research, there are concerns that should be optimized in future revisions. The current RQS is mainly focused on radiomics itself and ignores non-radiomics components during radiomics model/predictor development, such as blindness to outcomes and measurement, intervals between the index test and reference standard (in the case of MVI, the time between imaging and liver resection), and the influence of sample size and enrollment of study subjects. All these factors may also introduce bias. Under this context, the tool of QUADAS-2 can serve as a vital supplement to RQS when evaluating the quality of radiomics research. Most of the studies reported in this systematic search showed a low or unclear risk in the four domains of risk of bias evaluation. The missing or unclear parts observed using the RQS and QUADAS-2 tools were obvious, which implies that these tools might not be so well known or adopted. Future researchers will ideally apply the RQS or QUADAS-2 as a checklist to improve the quality of their reports. In fact, a specified checklist, i.e., CLAIM (Checklist for Artificial Intelligence in Medical Imaging) for artificial intelligence research [47], and a general guideline for diagnostic/prognostic prediction, i.e., TRIPOD (Transparent Reporting of a multivariable prediction model for Individual Prognosis Or Diagnosis) [15], have already been proposed.

This systematic review has some limitations. Firstly, high-level evidence from studies with a prospective design and an independent external validation cohort is lacking, so a definitive and convincing conclusion about the efficacy of the radiomics model for MVI prediction cannot be drawn. Secondly, we did not synthesize the performance metrics of the prediction model, given the high methodological heterogeneity of each study. Therefore, model performance comparisons between semantic-feature-based models and radiomics models, between CT-based models and MRI-based models, and between dilated-VOI-based models and non-dilated models could not be performed. Thirdly, we did not evaluate the specific radiomics features shared among different models due to the variability of imaging modalities and the extraction software used.

## 5. Conclusions

Even though current studies were preliminary and the methodological quality was insufficient, the radiomics model has the potential to provide an accurate and effective tool to preoperatively predict MVI presence in patients with HCC. Future prospective studies with an external validation cohort in accordance with a standardized radiomics workflow and reporting norms are expected to supply a reliable, reproducible, and accurate radiomics model for clinical implementation.

## Figures and Tables

**Table 1:** Study and patient characteristics.

First Author	Year	Study Design	No. of Patients (Train vs. Test Cohort)	Independent Validation Cohort	Age (Mean/Median)	Gender (M/F, %)	Indication	MVI Incidence
Jian Zheng [20]	2017	R#	120 (NA)	No	70	73/27	HCC	44%
Jie Peng [21]	2018	R	304 (184:120)	No	53 vs. 55 ^†^	85/15	HCC (solitary)	66%
Xiaohong Ma [22]	2018	R	157 (110:47)	No	53 vs. 55 ^†^	85/15	HCC (≤6 cm, solitary)	35%
ShiTing Feng [23]	2019	R	160 (110:50)	No	54.8	91/9	HCC	38.8%
Ming Ni [24]	2019	R	206 (148:58)	No	57 vs. 59 ^†^	NA	HCC (>1 cm)	42.7%
Rui Zhang [25]	2019	R	267 (194:73)	No	57.9	86/14	HCC (solitary)	33.7%
Yong-Jian Zhu [26]	2019	R	142 (99:43)	No	57	87/13	HCC (<5 cm, solitary)	37.3%
Giacomo Nebbia [27]	2020	R	99 (NA)	No	51 vs. 54 (MVI vs. non-MVI)	84/16	HCC	61.6%
Qiu-ping Liu [28]	2020	R	494 (346:148)	No	NA	84/16	HCC	30.2%
Xiuming Zhang [29]	2020	R	637 (451:111)	Yes (75, external)	57.5 vs. 56.2 vs. 60.7 ^§^	86/14	HCC	40%
Yi-quan Jiang [30]	2020	R	405 (324:81)	No	48.5	85/15	HCC	54.3%
Mu He [31]	2020	R	163 (101:44)	Yes (18, internal)	50.0 vs. 47.5 vs. 52.0 ^§^	82/18	HCC	67.5%
Huan-Huan Chong [32]	2021	R	356 (250:106)	No	54.2	85/15	HCC (≤5 cm)	25.3%
Yidi Chen [33]	2021	R	269 (188:81)	No	51.5	81/19	HCC	41.3%
Youcai Li [34]	2021	R	80 (50:30)	No	NA	91/9	HCC (BCLC 0/A)	45%
Danjun Song [35]	2021	R	601 (461:140)	No	56.5	82/18	HCC (solitary)	37.40%
Houjiao Dai [36]	2021	R	69 (LOOCV)	No	52.7	96/4	HCC (solitary)	42.0%
Peng Liu [37]	2021	R	185 (124:61)	No	54 vs. 52 ^†^	84/26	HCC (≤5 cm, solitary)	34.1%
Shuai Zhang [38]	2021	R	130 (91:39)	No	57.8 vs. 58.6 ^†^	68/32	HCC (>1 cm)	61.5%
Wanli Zhang [39]	2021	R	111 (88:23)	No	NA	88/12	HCC	51.4%
Xiang-pan Meng [40]	2021	R	402 (300:102)	No	57 vs. 57 ^†^	85/15	HCC (solitary)	40%
Yang Zhang [41]	2021	R	195 (136:59)	No	57.7	88/12	HCC (≤5 cm)	56.4%

Note: #, respective study; ^†^, train vs. test cohort; ^§^, train vs. test vs. validation cohort; BCLC, the Barcelona Clinic Liver Cancer staging system; HCC, hepatocellular carcinoma; LOOCV, leave-one-out cross validation; MVI, microvascular invasion; NA, not applicable.

**Table 2:** Characteristics of the radiomics research for microvascular invasion (MVI) prediction.

Study ID	Imaging Modality	Phase/Sequence	Segmentation	Extension of VOI	Feature Selection	Number of Imaging Features Included in the Model	Event/Feature Ratio during Model Development	Clinical Variables for Modeling	AUC in Test Cohort
Zheng 2017	CE-CT	PVP	2D; auto	Yes (5-pixel)	univariable logistic regression	21	1.6	AFP, tumor size, hepatitis	NA
Peng 2018	CE-CT	AP, PVP, DP	3D; semi-auto	No	LASSO	8	15.9	AFP, non-smooth tumor margin, internal arteries, hypoattenuating halos	0.84
Ma 2018	CE-CT	AP, PVP, DP	3D; manually	No	ICC/CCC, LASSO	7	5.3	Age, tumor size, hepatitis B	0.80
Feng 2019	Gd-EOB-DTPA MRI	HBP	3D; manually	Yes (10 mm)	LASSO	10	4.2	NA	0.84
Ni 2019	CE-CT	PVP	2D; manually	No	LASSO, neighbourhood rough set, PCA	Unclear	NA	NA	NA
R. Zhang 2019	Multimodel MRI	AP, PVP, EP, T1, T2, DWI	3D; manually	Yes (10 mm)	mRMR	12	5.3	AFP, arterial peritumoral enhancement	0.86
Zhu 2019	CE-MRI	AP, PVP	3D; manually	No	Kruskal-Wallis test; Pearson correlation	4	9.3	AFP, tumor size, differentiation	0.79
Nebbia 2020	CE-MRI	AP, PVP, T1, T2, DWI	3D; manually	Yes (10 pixel)	LASSO	17	3.6	NA	NA
Q. Liu 2020	CE-CT	AP, PVP	3D;semiauto	No	ICC, RF	28	5.3	NA	0.79
X. Zhang 2020	CE-CT	DP	3D; manually	Yes (10 mm)	LASSO	44	4.0	Age, AFP	0.80 (0.80 in the validation cohort)
Jiang 2020	CE-CT	AP, PVP, DP	3D; manually	Yes (10 mm)	Xgboost/3D-CNN	Unclear	NA	AFP	0.91
He 2020	CE-CT	PVP	3D; auto	No	LASSO	2	35.5	AFP, neutrophilic granulocytes, hemoglobin	0.71 (0.84 in the validation cohort)
Chong 2021	Gd-EOB-DTPA MRI	AP, PVP, HBP, DWI	3D; manually	Yes (5 mm,10 mm, 50%)	ICC, LASSO, Univariate Feature Selection	74	0.9	AFP, TBIL, capsule enhancement, peritumoral enhancement	0.92
Chen 2021	Gd-EOB-DTPA MRI	AP, PVP, HBP, T1, T2, DWI	3D; manually	No	LASSO	21	NA	NA	0.94
Li 2021	PET/CT	[18F]FDG PET/CT	3D; semiauto	No	LASSO	11	0.7	NA	0.69
Song 2021	CE-MRI	AP, PVP, DP, T1,T2, ADC, DWI	3D; manually	No	PCA, analysis of variance	Unclear	NA	NA	0.73
Dai 2021	Gd-EOB-DTPA MRI	AP, PVP, HBP, T1	3D; manually	No	LASSO-RFE, LASSO, mRMR, SVM-RFE	5	5.8	NA	0.90 (LOOCV)
P. Liu 2021	CE-CT	AP	3D; manually	No	ICC, LASSO	10	4.2	NA	0.75
Sh. Zhang 2021	Gd-EOB-DTPA MRI	HBP (5, 10, 15 min)	3D; manually	No	LASSO	14	NA	NA	NA
W. Zhang 2021	CE-CT	EAP, LAP, PVP, EP	3D; manually	2, 4, 6, 8, 10, 12, 14 mm	15 methods (Fisher score, t score, etc.)	Unclear	NA	NA	0.81
Meng 2021	Multiparametric MRI & multiphasic CT	AP, PVP, T2, DWI (MRI)/AP, PVP (CT)	3D; manually	Yes (3 mm)	ICC, univariate analysis, feature reduction, LASSO	16/16	8.1/1.9 ^#^	NA	0.80
Y. Zhang 2021	Multiparametric MRI	AP, PVP, DP, T2, DWI, ADC	3D; manually	No	ICC, analysis of variance, Mann-Whitney U-test, correlation analysis, LASSO	47	1.6	Age, AFP, tumor size	0.84

Note: # for CT and MRI model respectively. 3D-CNN, 3D-convolutional neural network; ADC, apparent diffusion coefficient; AFP, alpha-fetoprotein; AP, arterial phase; CCC, concordance correlation coefficient; CE-CT, contrast enhanced-CT; CE-MRI, contrast enhanced-MRI; DP, delay phase; DWI, diffusion-weighted imaging; EAP, early arterial phase; EP, equilibrium phase; HBP, hepatobiliary phase; ICC, interclass correlation coefficient; LASSO, least absolute shrinkage and selection operator; LOOCV, leave-one out cross validation; mRMR, minimum redundancy-maximum relevance; NA, not applicable; PCA, principal component analysis; PVP, portal vein phase; RFE, recursive feature elimination; SVM, Support vector machine; TBIL, total bilirubin; VOI, volume of interest.

## Supplementary Materials

The following are available online at https://www.mdpi.com/article/10.3390/cancers13225864/s1, Table S1: Preferred Reporting Items for a Systematic Review and Meta-analysis of Diagnostic Test Accuracy Studies (PRISMA-DTA) Checklist. Table S2: Literature searching strategy in the PubMed, Web of Science, Embase, and Cochrane library databases. Table S3: Description of the Radiomics Quality Score (RQS) tool. Table S4: Description of the revised Quality Assessment of Diagnostic Accuracy Studies (QUADAS-2) tool. Table S5: Methodological quality assessment of each study by the RQS tool. Table S6: Methodological quality assessment of each study by QUADAS-2. Table S7: Characteristics of the radiomics studies on the prediction of microvascular invasion in hepatocellular carcinoma.
