# Supplementary material for: Radiomics Models for Predicting Microvascular Invasion in Hepatocellular Carcinoma: A Systematic Review and Radiomics Quality Score Assessment
Source: Cancers (Basel). 2021 Nov 22;13(22):5864. doi: 10.3390/cancers13225864 (PMC8616379; doi:10.3390/cancers13225864)
Supplement: Supplementary file 1 [file cancers-13-05864-s001.zip › cancers-1443456-SUPPLEMENTARY .pdf]

Supplementary Materials

Radiomics models for predicting microvascular invasion in hepatocellular carcinoma: A systematic review and Radiomics Quality Score assessment

Qiang Wang*, Changfeng Li, Jiaxing Zhang, Xiaojun Hu, Yingfang Fan, Kuansheng Ma, Ernesto Sparrelid, Torkel B. Brismar

Contents

Table S1: Preferred Reporting Items for a Systematic Review and Meta-analysis of Diagnostic Test Accuracy Studies (PRISMA-DTA) Checklist

Table S2. literature searching strategy in databased of PubMed, Web of science, Embase and Cochrane library.

Table S3. Description of the radiomics quality score (RQS) tool.

Table S4. Description of the revised Quality Assessment of Diagnostic Accuracy Studies (QUADAS-2) tool.

Table S5. Methodological quality assessment of each study by RQS tool.

Table S6. Risk of bias and application concerns assessment of each study by the QUADAS-2 tool.

Table S7. Characteristics of the radiomics studies in prediction of microvascular invasion in hepatocellular carcinoma

Table s1. Preferred Reporting Items for a Systematic Review and Meta-analysis of Diagnostic Test Accuracy Studies (PRISMA-DTA) Checklist

Section/topic	#	PRISMA-DTA Checklist Item	Reported on page #
**TITLE / ABSTRACT**			
Title	1	Identify the report as a systematic review (+/- meta-analysis) of diagnostic test accuracy (DTA) studies.	P1
Abstract	2	Abstract: See PRISMA-DTA for abstracts.	P2-3
**INTRODUCTION**			
Rationale	3	Describe the rationale for the review in the context of what is already known.	P3
Clinical role of index test	D1	State the scientific and clinical background, including the intended use and clinical role of the index test, and if applicable, the rationale for minimally acceptable test accuracy (or minimum difference in accuracy for comparative design).	P3-4
Objectives	4	Provide an explicit statement of question(s) being addressed in terms of participants, index test(s), and target condition(s).	P4
**METHODS**			
Protocol and registration	5	Indicate if a review protocol exists, if and where it can be accessed (e.g., Web address), and, if available, provide registration information including registration number.	P4
Eligibility criteria	6	Specify study characteristics (participants, setting, index test(s), reference standard(s), target condition(s), and study design) and report characteristics (e.g., years considered, language, publication status) used as criteria for eligibility, giving rationale.	P5
Information sources	7	Describe all information sources (e.g., databases with dates of coverage, contact with study authors to identify additional studies) in the search and date last searched.	P5
Search	8	Present full search strategies for all electronic databases and other sources searched, including any limits used, such that they could be repeated.	P5
Study selection	9	State the process for selecting studies (i.e., screening, eligibility, included in systematic review, and, if applicable, included in the meta-analysis).	P5
Data collection process	10	Describe method of data extraction from reports (e.g., piloted forms, independently, in duplicate) and any processes for obtaining and confirming data from investigators.	P5-6
Definitions for data extraction	11	Provide definitions used in data extraction and classifications of target condition(s), index test(s), reference standard(s) and other characteristics (e.g. study design, clinical setting).	P6-7
Risk of bias and applicability	12	Describe methods used for assessing risk of bias in individual studies and concerns regarding the applicability to the review question.	P7
Diagnostic accuracy measures	13	State the principal diagnostic accuracy measure(s) reported (e.g. sensitivity, specificity) and state the unit of assessment (e.g. per-patient, per-lesion).	N.A
Synthesis of results	14	Describe methods of handling data, combining results of studies and describing variability between studies. This could include, but is not limited to: a) handling of multiple definitions of target condition. b) handling of multiple thresholds of test positivity, c) handling multiple index test readers, d) handling of indeterminate test results, e) grouping and comparing tests, f) handling of different reference standards	N.A
**Section/topic**	**#**	**PRISMA-DTA Checklist Item**	**Reported on page #**
Meta-analysis	D2	Report the statistical methods used for meta-analyses, if performed.	N.A
Additional analyses	16	Describe methods of additional analyses (e.g., sensitivity or subgroup analyses, meta-regression), if done, indicating which were pre-specified.	N.A
**RESULTS**			
Study selection	17	Provide numbers of studies screened, assessed for eligibility, included in the review (and included in meta-analysis, if applicable) with reasons for exclusions at each stage, ideally with a flow diagram.	P8
Study characteristics	18	For each included study provide citations and present key characteristics including: a) participant characteristics (presentation, prior testing), b) clinical setting, c) study design, d) target condition definition, e) index test, f) reference standard, g) sample size, h) funding sources	P8-9
Risk of bias and applicability	19	Present evaluation of risk of bias and concerns regarding applicability for each study.	P12
Results of individual studies	20	For each analysis in each study (e.g. unique combination of index test, reference standard, and positivity threshold) report 2x2 data (TP, FP, FN, TN) with estimates of diagnostic accuracy and confidence intervals, ideally with a forest or receiver operator characteristic (ROC) plot.	N.A
Synthesis of results	21	Describe test accuracy, including variability; if meta-analysis was done, include results and confidence intervals.	N.A
Additional analysis	23	Give results of additional analyses, if done (e.g., sensitivity or subgroup analyses, meta-regression; analysis of index test: failure rates, proportion of inconclusive results, adverse events).	N.A
**DISCUSSION**			
Summary of evidence	24	Summarize the main findings including the strength of evidence.	P20
Limitations	25	Discuss limitations from included studies (e.g. risk of bias and concerns regarding applicability) and from the review process (e.g. incomplete retrieval of identified research).	P24
Conclusions	26	Provide a general interpretation of the results in the context of other evidence. Discuss implications for future research and clinical practice (e.g. the intended use and clinical role of the index test).	P24
**FUNDING**			
Funding	27	For the systematic review, describe the sources of funding and other support and the role of the funders.	P25

Adapted From: McInnes MDF, Moher D, Thombs BD, McGrath TA, Bossuyt PM, The PRISMA-DTA Group (2018). Preferred Reporting Items for a Systematic Review and Meta-analysis of Diagnostic Test Accuracy Studies: The PRISMA-DTA Statement. JAMA. 2018 Jan 23;319(4):388-396. doi: 10.1001/jama.2017.19163.

For more information, visit: **www.prisma-statement.org**.

Table s2: literature searching strategies in PubMed, Web of science, Embase and Cochrane Library.

No.	Search Query for PubMed	Result
#1	"Carcinoma, Hepatocellular"[Mesh] OR "Liver Neoplasms"[Mesh:NoExp]	159,239
#2	(Hepatocellular[Title/Abstract] OR "liver cell"[Title/Abstract] OR "hepatic cell"[Title/Abstract]) AND carcinoma*[Title/Abstract]	100,429
#3	hepatocarcinoma*[Title/Abstract] OR hepatoma*[Title/Abstract] OR "liver carcinoma"[Title/Abstract] OR HCC[Title/Abstract]	92,996
#4	**#1 OR #2 OR #3**	213,161
#5	"Microvessels"[Mesh] AND "Neoplasm Invasiveness"[Mesh]	339
#6	(microvascular*[Title/Abstract] OR micro-vascular*[Title/Abstract] OR microvessel*[Title/Abstract] OR microscop*[Title/Abstract] OR microvasculatur*[Title/Abstract]) AND (Portal*[Title/Abstract] OR Invasion*[Title/Abstract] OR Invasiveness[Title/Abstract])	16,133
#7	**#5 OR #6**	16,262
#8	textural* OR texture* OR radiomics* OR radiomic* OR histogram*	64,401
#9	**#4 AND #7 AND #8**	43

No.	Search Query for Web of science	Result
#1	TS=(((Hepatocellular OR "liver cell" OR "hepatic cell") AND carcinoma*) OR hepatocarcinoma* OR hepatoma* OR "liver carcinoma" OR HCC)	185,236
#2	TS=((microvascular* OR micro-vascular* OR microvessel* OR microscop* OR microvasculatur*) AND (Portal* OR Invasion* OR Invasiveness))	17,884
#3	TS = (textural* OR texture* OR radiomics* OR radiomic* OR histogram*)	324,738
#4	**#1 AND #2 AND #3 AND LANGUAGE: (English)**	77

No.	Search Query for Embase	Result
#1	'liver cell carcinoma'/exp OR 'liver cancer'/de	203,470
#2	((hepatocellular OR 'liver cell' OR 'hepatic cell') NEAR/6 carcinoma*):ab,ti,kw	144,801
#3	(hepatocarcinoma* OR hepatoma* OR 'liver carcinoma' OR HCC):ab,ti,kw	135,756
#4	**#1 OR #2 OR #3**	250,017
#5	'microvasculature'/exp AND 'tumor invasion'/exp	936
#6	((microvascular* OR micro-vascular* OR microvessel* OR microscop* OR microvasculatur*) NEAR/6 (Portal* OR invasion* OR Invasiveness)):ab,ti,kw	5,188
#7	**#5 OR #6**	5,953
#8	'radiomics'/exp OR 'texture'/exp OR ‘histogram'/exp	22,691
#9	‘radiomic’:ti,ab,kw OR ‘radiomics':ti,ab,kw OR ‘textural':ti,ab,kw OR ‘texture':ti,ab,kw OR ‘histogram’:ti,ab,kw	60,239
#10	**#8 OR #9**	70,697
#11	**#4 AND #7 AND #10**	59

No.	Search Query for Cochrane library	Result
#1	MeSH descriptor: [Carcinoma, Hepatocellular] OR MeSH descriptor: [Liver Neoplasms]	3074
#2	((hepatocellular OR 'liver cell' OR 'hepatic cell') NEAR/6 carcinoma*):ab,ti,kw	24707
#3	(hepatocarcinoma* OR hepatoma* OR 'liver carcinoma' OR HCC):ab,ti,kw	7368
#4	**#1 OR #2 OR #3**	27087
#5	MeSH descriptor: [Microvessels] OR MeSH descriptor: [Neoplasm Invasiveness]	1252
#6	((microvascular* OR micro-vascular* OR microvessel* OR microscop* OR microvasculatur*) NEAR/6 (Portal* OR invasion* OR Invasiveness)):ab,ti,kw	110
#7	**#5 OR #6**	1356
#8	‘radiomic’:ti,ab,kw OR ‘radiomics':ti,ab,kw OR ‘textural':ti,ab,kw OR ‘texture':ti,ab,kw OR ‘histogram’:ti,ab,kw	2231
#9	**#4 AND #7 AND #8**	9

Table s3: Description of the radiomics quality score (RQS) tool.

	Criteria	Points
**1**	**Image protocol quality** – well-documented image protocols (e.g., contrast, slice thickness, energy, etc.) and/or usage of public image protocols allow reproducibility/ replicability	+1 (if protocols are well-documented)<br>+1 (if public protocol is used)
**2**	**Multiple segmentations** – possible actions are: segmentation by different physicians/ algorithms/software, perturbing segmentations by (random) noise, segmentation at different breathing cycles. Analyze feature robustness to segmentation variabilities	+1
**3**	**Phantom study on all scanners** – detect inter-scanner differences and vendor-dependent features. Analyze feature robustness to these sources of variability	+1
**4**	**Imaging at multiple time points** – collect individuals' images at additional time points. Analyze feature robustness to temporal variabilities (e.g., organ movement, organ expansion/shrinkage).	+1
**5**	**Feature reduction** or adjustment for multiple testing – decreases the risk of overfitting. Overfitting is inevitable if the number of features exceeds the number of samples. Consider feature robustness when selecting features	+3 (if neither measure is implemented)<br>+3 (if either measure is implemented)
**6**	**Multivariable analysis** with non radiomic features (e.g., EGFR mutation) – is expected to provide a more holistic model. Permits correlating/inferencing between radiomics and non radiomics features	+1
**7**	Detect and discuss **biological correlates** – demonstration of phenotypic differences (possibly associated with underlying gene–protein expression patterns) deepens understanding of radiomics and biology	+1
**8**	**Cut-off analyses** – determine risk groups by either the median, a previously published cut-off or report a continuous risk variable. Reduces the risk of reporting overly optimistic results	+1
**9**	**Discrimination statistics** – report discrimination statistics (e.g., C-statistic, ROC curve, AUC) and their statistical significance (e.g., p-values, confidence intervals). One can also apply resampling method (e.g., bootstrapping, cross-validation)	+1 (if a discrimination statistic and its statistical significance are reported)<br>+1 (if also an resampling method technique is applied)
**10**	**Calibration statistics** – report calibration statistics (e.g., Calibration-in-the-large/slope, calibration plots) and their statistical significance (e.g., p-values, confidence intervals). One can also apply resampling method (e.g., bootstrapping, cross-validation)	+1 (if a calibration statistic and its statistical significance are reported)<br>+1 (if also an resampling method technique is applied)
**11**	**Prospective study** registered in a trial database – provides the highest level of evidence supporting the clinical validity and usefulness of the radiomics biomarker	+7 (for prospective validation of a radiomics signature in an appropriate trial)

**12**	**Validation** – the validation is performed without retraining and without adaptation of the cut-off value, provides crucial information with regard to credible clinical performance	-5 (if validation is missing)<br>+2 (if validation is based on a dataset from the same institute)<br>+3 (if validation is based on a dataset from another institute)<br>+4 (if validation is based on two datasets from two distinct institutes)<br>+4 (if the study validates a previously published signature)<br>+5 (if validation is based on three or more datasets from distinct institutes)<br>*Datasets should be of comparable size and should have at least 10 events per model feature.
**13**	**Comparison to 'gold standard'** – assess the extent to which the model agrees with/is superior to the current 'gold standard' method (e.g., TNM-staging for survival prediction). This comparison shows the added value of radiomics	+2
**14**	**Potential clinical utility** – report on the current and potential application of the model in a clinical setting (e.g., decision curve analysis)	+2
**15**	**Cost-effectiveness analysis** – report on the cost-effectiveness of the clinical application (e.g., quality adjusted life years generated)	+1
**16**	**Open science and data** – make code and data publicly available. Open science facilitates knowledge transfer and reproducibility of the study	+1 (if scans are open source)<br>+1 (if region of interest segmentations are open source)<br>+1 (if code is open source)<br>+1 (if radiomics features are calculated on a set of representative ROIs and the calculated features + representative ROIs are open source
	**Total points (36 = 100%)**	

Source: Lambin P, Leijenaar RTH, Deist TM, et al. Radiomics: the bridge between medical imaging and personalized medicine. Nat Rev Clin Oncol. 2017 Dec;14(12):749-762. doi: 10.1038/nrclinonc.2017.141. Epub 2017 Oct 4. PMID: 28975929.

Table s4: Description of the revised Quality Assessment of Diagnostic Accuracy Studies (QUADAS-2) tool

Domain	Patient selection	Index test	Reference standard	Flow and timing
**Description**	Describe methods of patient selection; Describe included patients (previous testing, presentation, intended use of index test, and setting).	Describe the index test and how it was conducted and interpreted.	Describe the reference standard and how it was conducted and interpreted.	Describe any patients who did not receive the index tests or reference standard or who were excluded from the 2*2 table; Describe the interval and any interventions between index tests and the reference standard.
**Signalling questions (yes, no, or unclear)**	Was a consecutive or random sample of patients enrolled?<br>Was a case-control design avoided?<br>Did the study avoid inappropriate exclusions?	Were the index test results interpreted without knowledge of the results of the reference standard?<br>If a threshold was used, was it prespecified?	Is the the reference standard likely to correctly classify the target condition?<br>Were the reference standard results interpreted without knowledge of the results of the index test?	Was there an appropriate interval between index test and reference standard?<br>Did all patients receive a reference standard?<br>Did all patients receive the same reference standard?<br>Were all patients included in the analysis?
**Risk of bias (high, low, or unclear)**	Could the selection of patients have introduced bias?	Could the conduct or interpretation of the index test have introduced bias?	Could the reference standard, its conduct, or its interpretation have introduced bias?	Could the patient flow have introduced bias?
**Concerns about applicability (high, low, or unclear)**	Are there concerns that the included patients do not match the review question?	Are there concerns that the index test, its conduct, or its interpretation differ from the review question?	Are there concerns that the target condition as defined by the reference standard does not match the review question?	-

Source: Whiting PF, Rutjes AW, Westwood ME, et al; QUADAS-2 Group. QUADAS-2: a revised tool for the quality assessment of diagnostic accuracy studies. Ann Intern Med. 2011 Oct 18;155(8):529-36. doi: 10.7326/0003-4819-155-8-201110180-00009. PMID: 22007046.

Table s5. Methodological quality assessment of each study by the RQS tool

Study ID	Image protocol quality	Multiple segmentations	Phantom study	Imaging at multiple time points	Feature reduction	Multivariable analysis with non radiomics features	Biological correlates	Cut-off analyses	Discrimination statistics	Calibration statistics	Prospective study	Validation	Comparison to ‘gold standard’	Potential clinical utility	Cost-effectiveness analysis	Open science and data	Total points
**Zheng2017**	0	0	0	0	3	1	0	0	1	0	0	0	0	0	0	0	5 (14%)
**Peng2018**	1	0	0	0	3	1	0	1	2	1	0	2	2	2	0	0	15 (42%)
**Ma2018**	1	1	0	0	3	1	0	0	2	1	0	2	0	2	0	0	13 (36%)
**Feng2019**	1	1	0	0	3	0	0	0	2	0	0	2	2	0	0	0	11 (31%)
**Ni2019**	1	0	0	0	3	0	0	0	2	0	0	2	0	2	0	0	10 (28%)
**R. Zhang2019**	1	1	0	0	3	1	0	0	1	1	0	2	2	2	0	0	14 (39%)
**Zhu2019**	1	0	0	0	3	1	0	1	1	0	0	2	0	0	0	0	9 (25%)
**Nebbia2020**	1	0	0	0	3	0	0	0	2	0	0	0	0	0	0	0	6 (17%)
**Q. Liu 2020**	1	1	0	0	3	0	0	0	1	0	0	2	0	0	0	0	8 (22%)
**X. Zhang2020**	1	0	0	0	3	1	0	0	2	1	0	3	0	0	0	0	11 (31%)
**Jiang2020**	1	0	0	0	0	1	0	0	1	0	0	2	2	0	0	0	7 (19%)
**He2020**	1	0	0	0	3	1	0	0	2	1	0	2	0	2	0	0	12 (33%)
**Chong2021**	1	1	0	0	0	1	0	1	2	1	0	2	2	2	0	0	13 (36%)
**Chen2021**	1	0	0	0	3	0	0	0	2	0	0	2	2	0	0	0	10 (28%)
**Li2021**	1	0	0	0	3	0	0	1	1	1	0	2	0	2	0	0	11 (31%)
**Song2021**	1	0	0	0	3	0	0	0	1	0	0	2	0	0	0	0	7 (19%)
**Dai2021**	1	0	0	0	3	0	0	0	2	0	0	2	0	0	0	0	8 (22%)
**P. Liu2021**	1	1	0	0	3	0	0	0	2	0	0	2	2	0	0	0	11 (31%)
**Sh. Zhang2021**	1	1	0	0	3	0	0	0	2	0	0	2	2	2	0	0	13 (36%)
**W. Zhang2021**	1	1	0	0	3	0	0	0	2	0	0	2	0	0	0	0	9 (25%)
**Meng2021**	1	1	0	1	3	0	0	0	2	1	0	2	2	0	0	0	13 (36%)
**Y. Zhang2021**	1	1	0	0	3	1	0	1	2	1	0	2	0	2	0	0	14 (39%)

Table s6. Risk of bias and application concerns assessment of each study by the QUADAS-2 tool

Study ID	Risk of Bias				Applicability Concerns		
	Patient selection	Index test	Reference standard	Flow and timing	Patient selection	Index test	Reference standard
**Zheng2017**	Low	Low	Unclear	Unclear	Low	Unclear	Unclear
**Peng2018**	Low	High	Unclear	Unclear	Low	Low	Unclear
**Ma2018**	Unclear	Unclear	Low	Unclear	High	Low	Low
**Feng2019**	Low	Unclear	Low	Low	Low	Rad score	Low
**Ni2019**	Unclear	Unclear	Unclear	Unclear	Low	Unclear	Unclear
**R. Zhang2019**	Low	Low	Low	Low	Low	Low	Low
**Zhu2019**	Low	High	Low	Low	High	Low	Low
**Nebbia2020**	Unclear	Unclear	Low	Low	Unclear	Unclear	Low
**Q. Liu 2020**	Unclear	Unclear	Low	Low	Unclear	Unclear	Low
**X. Zhang2020**	Low	Unclear	Low	Low	Low	Low	Low
**Jiang2020**	Unclear	Unclear	Low	High	Unclear	Unclear	Low
**He2020**	Unclear	Unclear	Low	Low	Low	Low	Low
**Chong2021**	Low	High	Low	Low	High	Low	Low
**Chen2021**	Low	Low	Low	Unclear	Low	Unclear	Low
**Li2021**	High	Unclear	Low	Low	Low	Low	Low
**Song 2021**	Low	Unclear	Low	Low	Low	Unclear	Low
**Dai2021**	Low	Unclear	Unclear	Unclear	Low	Unclear	Unclear
**P. Liu2021**	Low	Low	Unclear	Unclear	High	Low	Unclear
**Sh. Zhang2021**	Low	Low	Unclear	Unclear	Low	Unclear	Unclear
**W. Zhang2021**	Low	Low	Low	Low	Low	Unclear	Low
**Meng2021**	Low	Low	Low	Low	Low	Unclear	Low
**Y. Zhang2021**	Low	High	Unclear	Unclear	High	Unclear	Unclear

Table s7. Characteristics of the radiomics research for microvascular invasion prediction

Study ID	Country	Consecutive patient	Interval between imaging scanning and liver resection	No. of readers	Blindness to outcomes	MVI criteria	VOI software	Resampling	ICC evaluation (threshold)	Feature extraction software	No. of extraced feature (each VOI/ROI)	Calibration analysis	Decision curve analysis	AUC of radiomics model vs conventional radiological model
**Zheng2017**	USA	Yes	3 months	2	Yes	Unclear	Scout Liver	Unclear	No	Matlab	166	No	No	NA
**Peng2018**	China	Yes	1 week	2	Yes	Unclear	IBEX	Unclear	No	IBEX(Matlab)	490	Yes	Yes	Rad-model: TTPVI: RVI=0.85: 0.72:0.65
**Ma2018**	China	Unclear	Unclear	2	Unclear	Yes	ITK-SNAP	Unclear	Yes(0.75)	Matlab	647	Yes	Yes	NA
**Feng2019**	China	Yes	1 month	3	Unclear	Yes	ITK-SNAP	Unclear	Yes	Artificial intelligence kit,GE	1044	No	No	0.84 vs 0.62(accuracy)
**Ni2019**	China	Unclear	1 month	2	Unclear	Unclear	NA	Unclear	No	Artificial intelligence kit,GE	1044	No	Yes	NA
**R. Zhang2019**	China	Yes	1 month	2	Yes	Yes	ITK-SNAP	Yes	Yes	Matlab	2932	Yes	Yes	0.82 vs 0.72
**Zhu2019**	China	Yes	15 days	2	Yes	Yes	Omni-kinetics	Unclear	No	Omni-kinetics	58	No	No	NA
**Nebbia2020**	China	Unclear	1 week	2	Unclear	Yes	Unknown	Unclear	No	pyradiomics	100	No	No	NA
**Q. Liu2020**	China	Unclear	1 month	2	Unclear	Yes	in-house software(ONCO)	Unclear	Yes(0.9)	pyradiomics	1210	No	No	NA
**X. Zhang2020**	China	Yes	1 month	2	Unclear	Yes	ITK-SNAP	Yes	No	NA	798	Yes	No	NA
**Jiang2020**	China	Yes	2 months	3	Unclear	Yes	ITK-SNAP	Unclear	No	pyradiomics	1217	No	No	0.89 vs 0.88

**He2020**	China	Unclear	1 week	1	Unclear	Yes	Home-made(liver parenchyma)	Unclear	No	pyradiomics	1231	Yes	Yes	NA
**Chong2021**	China	Yes	1 month	2	Yes	Yes	ITK-SNAP	unclear	Yes(0.8)	pyradiomics	854	Yes	Yes	0.92 vs 0.88
**Chen2021**	China	Yes	Unclear	2	Yes	Yes	NA	Yes	No	pyradiomics	1395	No	No	0.94 vs 0.85
**Li2021**	China	Yes	2 weeks	1	Unclear	Yes	Lifex	Yes	No	Lifex	101	Yes	Yes	NA
**Song 2021**	China	Yes	1 month	4	Unclear	Yes	Unclear	Yes	No	pyradiomics	110	No	No	NA
**Dai2021**	China	Yes	1 month	2	Unclear	Unclear	ITK-SNAP	Yes	No	Matlab-based in house software	167	No	No	NA
**P. Liu2021**	China	Yes	Unclear	1	Yes	Unclear	3D-slicer	Unclear	Yes(0.75)	3D-slicer	1351	No	No	Rad-score: TTPVI: RVI=0.75: 0.52:0.53
**Sh. Zhang2021**	China	Yes	1 month	2	Yes	Unclear	IBEX	Unclear	Yes(0.8)	IBEX(Matlab)	1768	No	Yes	NA
**W. Zhang2021**	China	Yes	2 weeks	2	Yes	Yes	ITK-SNAP	Unclear	Yes(Dice similarity > 0.9)	pyradiomics	94	No	No	NA
**Meng2021**	China	Yes	4 weeks	3/2	Yes	Yes	3D Slicer	Yes	Yes(0.8)	pyradiomics	1288	Yes	No	CT: Rad-model vs R-R model = 0.81 vs 0.84; MRI: Rad-model vs R-R model = 0.83 vs 0.87
**Y. Zhang2021**	China	Yes	1 week	2	Yes	Unclear	ITK-SNAP	Yes	Yes(0.8)	Artificial intelligence kit,GE	396	Yes	Yes	NA

Note: AUC, area under the receiver operating characteristic curve; ICC, interclass correlation coefficient; MVI, microvascular invasion; NA, not available; Rad-model, radiomics model; R-R model, radiologic-radiomics model; ROI, region of interest; RVI, radiogenomic venous invasion; TTPVI, two-trait predictor of venous invasion; VOI, volume of interest
